# Association between supportive interventions and healthcare utilization and outcomes in patients on long-term prescribed opioid therapy presenting to acute healthcare settings: a systematic review and meta-analysis

**DOI:** 10.1186/s12873-020-00398-9

**Published:** 2021-01-29

**Authors:** Jean Deschamps, James Gilbertson, Sebastian Straube, Kathryn Dong, Frank P. MacMaster, Christina Korownyk, Lori Montgomery, Ryan Mahaffey, James Downar, Hance Clarke, John Muscedere, Katherine Rittenbach, Robin Featherstone, Meghan Sebastianski, Ben Vandermeer, Deborah Lynam, Ryan Magnussen, Sean M. Bagshaw, Oleksa G. Rewa

**Affiliations:** 1 Department of Critical Care Medicine, Faculty of Medicine and Dentistry, University of Alberta, 8440 112 St. NW, Critical Care Medicine 2-124E Clinical Sciences Building, Edmonton, Alberta T6G 2B7 Canada; 2 School of Medicine, Faculty of Medicine and Dentistry, University of Alberta, Edmonton, Alberta Canada; 3 Division of Preventive Medicine, Department of Medicine, University of Alberta, 5-30 University Terrace, 8303 – 112 St NW, Edmonton, Alberta T6G 2T4 Canada; 4 Department of Emergency Medicine, University of Alberta, 2J2.00 WC Mackenzie Health Sciences Centre, 8440 112 St NW, Edmonton, Alberta T6G 2R7 Canada; 5 Departments of Psychiatry and Pediatrics, University of Calgary, Strategic Clinical Network for Addictions and Mental Health 2888 Shaganappi Trail NW Calgary, Calgary, Alberta T3B 6A8 Canada; 6 Department of Family Medicine, University of Alberta, Suite 205 College Plaza, 8215 112 St NW, Edmonton, Alberta T6G 2C8 Canada; 7 Department of Family Medicine, Calgary Chronic Pain Center 1820 Richmond Road SW Calgary, Calgary, Alberta T2T 5C7 Canada; 8 Department of Anesthesia, University of Ottawa, Ottawa, Ontario Canada; 9 Department of Medicine, University of Ottawa, Ottawa, Ontario Canada; 10 Department of Anesthesia and Pain Management, Toronto General Hospital, University Health Network, Ottawa, Ontario Canada; 11 Transitional Pain Program, Toronto General Hospital, University Health Network, Ottawa, Ontario Canada; 12 Department of Critical Care Medicine, Queen’s University, Kingston, Ontario Canada; 13 Addiction & Mental Health Strategic Clinical Network, Alberta Health Services, Edmonton, Alberta Canada; 14 Department of Psychiatry, University of Alberta, Edmonton, Alberta Canada; 15 Department of Psychiatry, University of Calgary, Calgary, Canada; 16 Alberta Research Centre for Health Evidence, Department of Pediatrics, University of Alberta, Alberta SPOR SUPPORT Unit KT Platform, 4-486D Edmonton Clinical Health Academy, 11405 – 87 Avenue, Edmonton, Alberta T6G 1C9 Canada; 17 Knowledge Translation Platform, Alberta SPOR SUPPORT Unit Department of Pediatrics, University of Alberta, 362-B Heritage Medical Research Centre (HMRC), Edmonton, Canada; 18 Primary Health Care Information Network, Edmonton, Alberta Canada; 19 Critical Care Strategic Clinical Network, Foothills Medical Centre, ICU Administration – Ground Floor, McCaig Tower, 3134 Hospital Drive, Calgary, Alberta T2N 2T9 Canada

**Keywords:** Opioid, Addiction medicine, Substance-related disorders, Drug abuse, Hospital medicine

## Abstract

**Background:**

Long-term prescription of opioids by healthcare professionals has been linked to poor individual patient outcomes and high resource utilization. Supportive strategies in this population regarding acute healthcare settings may have substantial impact.

**Methods:**

We performed a systematic review and meta-analysis of primary studies. The studies were included according to the following criteria: 1) age 18 and older; 2) long-term prescribed opioid therapy; 3) acute healthcare setting presentation from a complication of opioid therapy; 4) evaluating a supportive strategy; 5) comparing the effectiveness of different interventions; 6) addressing patient or healthcare related outcomes. We performed a qualitative analysis of supportive strategies identified. We pooled patient and system related outcome data for each supportive strategy.

**Results:**

A total of 5664 studies were screened and 19 studies were included. A total of 9 broad categories of supportive strategies were identified. Meta-analysis was performed for the “supports for patients in pain” supportive strategy on two system-related outcomes using a ratio of means. The number of emergency department (ED) visits were significantly reduced for cohort studies (*n* = 6, 0.36, 95% CI [0.20–0.62], I^2^ = 87%) and randomized controlled trials (RCTs) (*n* = 3, 0.71, 95% CI [0.61–0.82], I^2^ = 0%). The number of opioid prescriptions at ED discharge was significantly reduced for RCTs (*n* = 3, 0.34, 95% CI [0.14–0.82], I^2^ = 78%).

**Conclusion:**

For patients presenting to acute healthcare settings with complications related to long-term opioid therapy, the intervention with the most robust data is “supports for patients in pain”.

**Supplementary Information:**

The online version contains supplementary material available at 10.1186/s12873-020-00398-9.

## Background

### Description of the condition

The opioid epidemic is a major public health problem across the world. While not initially recognized as a crisis, it has become a public health emergency that is largely believed to have begun in 1996 when OxyContin® was approved by the FDA [1]. Opioid prescription was initially thought to be the crux of the problem and multiple interventions were implemented in response over the years. Most of these interventions were focused on reducing access to opioid prescriptions with unforeseen and paradoxical consequences. Indeed, strictly targeting a reduction in opioid access ignored the complex socio-economic factors that impact opioid-related morbidity and mortality. As an example, reductions in prescription rates in Ontario (Canada) lead to a doubling of opioid mortality due to an increase in street opioids [2]. This shift in the nature of the opioid crisis to illicit opioid use has indeed been seen in multiple locations over the years and paralleled these interventions [3]. Considering such findings, illicit drug use and harm reduction related to their use has become the focus of most current efforts to limit the impact of the opioid crisis and the impact of opioid prescriptions has taken a backseat. However, it remains that there are patients on long-term opioids for chronic painful conditions without diagnosed or suspected opioid use disorder that are also at risk for complications. This group has ultimately been somewhat neglected amidst the extensive literature available on the more obvious high-risk populations. It is however quite clear that individuals on long-term opioid therapy are at risk of poor outcomes, including hospitalization, overdose and death from the use or management of their opioids [4, 5]. These poor outcomes invariably lead to acute healthcare presentations that have significant system level impacts on health services use including emergency department (ED) presentations, hospital and intensive care unit (ICU) admissions, as well important socio-economic consequences [6–10].

### Description of the intervention

Harm reduction strategies are defined as “any policy or program designed to reduce drug-related harm without requiring the cessation of drug use; these interventions may be targeted at the individual, the family, community or society [11–19].” Harm reduction strategies are traditionally thought of and applied to patients demonstrating high risk behaviors and the term has not typically been applied in relation to individuals on long-term opioid therapy without a diagnosed or suspected opioid use disorder. These individuals may still benefit from harm reduction strategies as they require these medications for their well-being but may still suffer harmful consequences from their use. Harm reduction strategies are however traditionally associated with opioid use disorder. There is thus ample evidence for harm reduction strategies in acute opioid overdoses that is lacking for individuals on long-term opioid therapy without opioid use disorder. Their impact may be substantial given the reported high risk of adverse events in these settings [7, 20–22]. As the strategies applying to this population may be different, we will instead refer to them as “supportive interventions” to limit confusion with strategies associated with opioid use disorder.

The most important complications as a result of opioid therapy most often lead to a presentation to an acute healthcare setting, defined as a setting in which “health system components, or care delivery platforms, are used to treat sudden, often unexpected, urgent or emergent episodes of injury and illness that can lead to death or disability without rapid intervention [23].” Examples of these include emergency departments, acute health care clinics, and hospital inpatient units. Accordingly, this may be the healthcare setting in which the most impactful supportive interventions may lie, and how this population can best be captured in studies. As such, the primary objective of this study was to identify the most effective supportive interventions for patients on long-term prescribed opioids presenting to acute care settings to decrease complications attributable to opioid use, to reduce avoidable health services use, and to improve outcomes.

## Methods

### Study design and registration

We performed a systematic review using the guidelines from Cochrane and the Centre for Reviews and Dissemination [24, 25], and reported according to the Preferred Reporting Items for Systematic Reviews and Meta-analyses (PRISMA) guideline and the Meta-analysis Of Observational Studies in Epidemiology (MOOSE) guidelines for observational studies [26, 27]. The study was registered with the PROSPERO (CRD42018088962 on 2018/02/19) International Prospective Register of Systematic Reviews (http://ww.crd.york.ac.uk/prospero).

### Criteria for considering studies for this review

#### Types of studies

We considered primary studies (i.e., randomized controlled trials (RCT), cohort studies, case-control studies) and secondary analyses or evidence syntheses (i.e., systematic reviews, meta-analyses). There were no language restrictions. We considered studies published after 1996 as this is when OxyContin® was introduced, and the current prescription opioid epidemic is believed to have largely began [1]. We excluded editorials, case series, case reports and narrative reviews.

#### Eligibility of individual studies

Studies were eligible for inclusion if they satisfied the following criteria:

- Patient related criteria:
  - Age 18 years or older.
  - Long-term opioid therapy, reflecting prescribed opioid more than 70% of days for at least 3 months [28].
  - Presentation to acute healthcare setting secondary for a presumed or confirmed complication of prescribed opioid therapy.
- Study-related criteria
  - Evaluating an intervention representing a harm reduction strategy.
  - Comparing the effectiveness of different interventions between each other or individual interventions compared to current care.
  - Addressing patient or healthcare system related outcomes (i.e. number of opioid prescriptions, repeat presentations to ED or acute healthcare, number of overdoses).

We excluded studies that specifically addressed patients with non-prescription opioid use, or prescription opioid use not obtained through healthcare professionals. We excluded studies of patients with opioid use disorder or misuse that was not attributable to an established chronic pain disorder or disease. These exclusions were chosen to target patients requiring long-term recurrent opioid use for medical conditions without a diagnosed or suspected disorder. Additionally, we eschewed the more stringent definition of long-term or chronic opioid therapy which usually is an “episode duration of 90+ days and 10+ opioid prescriptions or 120+ days supply of opioids dispensed [29].” We used a less stringent criteria to account for an expected smaller sample of population given the exclusions as mentioned. We will thus avoid the term “chronic opioid therapy” and use “long-term opioid therapy” as the former has stronger association with opioid use disorder and this may result in confusion for the reader.

### Search methods

The search strategy was developed and executed by an information specialist (RF) and was peer-reviewed by a second research librarian (Table 1). The information specialist searched electronic databases: Ovid MEDLINE (1946-), Ovid EMBASE (1996-), Cumulative Index to Nursing and Allied Health Literature (CINAHL) via EBSCOhost (1937-), and Wiley Cochrane Library (inception-), including the Cochrane Database of Systematic Reviews and the Cochrane Central Register of Controlled Trials (CENTRAL). We also searched reports from the National Information Center on Health Services Research and Health Care Technology (NICHSR) via the NICHSR ONESearch portal. Study records were also searched via the trial registry platform ClinicalTrials.gov, guidelines via the National Guidelines Clearinghouse, and meeting abstracts via the Conference Proceedings Citation Index database (Clarivate Analytics). The following search themes was used: 1) opioids; 2) long-term drug therapy; and 3) acute healthcare settings (emergency departments, acute care surgery, critical care, urgent care and short-term inpatient stabilization). We additionally scanned the reference list of relevant included studies for additional articles. Bibliographic records were exported to an EndNote X9 (Clarivate Analytics, Philadelphia, Pennsylvania). Databases were searched up to and including April 11, 2019.

**Table 1:** Search strategy

Database: Ovid MEDLINE(R) Epub Ahead of Print, In-Process & Other Non-Indexed Citations, Ovid MEDLINE(R) Daily and Ovid MEDLINE(R) 1946 to Present	
1 exp. Narcotics/ (111132)	
2 actiq*.tw,kf. (27)	
3 carfentan*.tw,kf. (243)	
4 codeine*.tw,kf. (4872)	
5 demerol*.tw,kf. (231)	
6 (dihydro-morph* or dihydromorph*).tw,kf. (451)	
7 dilaudid*.tw,kf. (69)	
8 dur?gesic*.tw,kf. (84)	
9 fentanyl*.tw,kf. (16667)	
10 fentora*.tw,kf. (9)	
11 heroin.tw,kf. (12893)	
12 (hydro-codone* or hydrocodone*).tw,kf. (858)	
13 (hydro-morphone* or hydromorphone*).tw,kf. (1359)	
14 morphine*.tw,kf. (47330)	
15 narcotic*.tw,kf. (14412)	
16 lorcet*.tw,kf. (5)	
17 lortab*.tw,kf. (6)	
18 opiate*.tw,kf. (23769)	
19 opioid*.tw,kf. (73603)	
20 (oxy-codone* or oxycodone*).tw,kf. (2670)	
21 (oxy-contin* or oxycontin*).tw,kf. (226)	
22 percocet*.tw,kf. (58)	
23 percodan*.tw,kf. (14)	
24 pethidine*.tw,kf. (2304)	
25 phentanyl*.tw,kf. (119)	
26 sublimaze*.tw,kf. (22)	
27 vicodin*.tw,kf. (56)	
28 or/1–27 [Combined MeSH & text words for opioids] (186296)	
29 Addiction Medicine/ (4)	
30 Behavior, Addictive/ (7744)	
31 exp. “Chemical and Drug Induced Liver Injury”/ (26678)	
32 Drug abuse/ (87805)	
33 exp. Drug Misuse/ (10703)	
34 Drug Overdose/ (9457)	
35 Neurotoxicity Syndromes/ (4428)	
36 exp. Opioid-Related Disorders/ (22304)	
37 Poisoning/ (21631)	
38 Psychoses, Substance-Induced/ (5082)	
39 Self-Injurious Behavior/ (6447)	
40 Street Drugs/ae [adverse effects] (1421)	
41 Substance-Related Disorders/ (87805)	
42 Substance Withdrawal Syndrome/ (20325)	
43 ((abus* or addict* or chronic* or depend* or disorder* or intoxicat* or mis-us* or misus* or over-dos* or overdos* or poison* or withdrawal*) adj3 (drug* or fentanyl* or heroin* or narcotic* or opiate* or opioid* or oxy-co* or oxyco* or morphine*)).tw,kf. (97309)	
44 ((drug* or substance* or toxic*) adj2 psycho*).tw,kf. (18771)	
45 or/29–44 [Combined MeSH & text words for chronic drug use] (263106)	
46 Burn Units/ (2227)	
47 Coronary Care Units/ (4202)	
48 exp. Critical Care/ (51242)	
49 Critical Care Nursing/ (1223)	
50 Emergency Medicine/ (11989)	
51 Emergency Nursing/ (6602)	
52 exp. Perioperative Care/ and (acute* or critical* or emergenc* or intensiv* or trauma* or urgent*).mp. (19203)	
53 Hospital Medicine/ (119)	
54 exp. Hospitals/ and (acute* or critical* or emergenc* or intensiv* or trauma* or urgent*).mp. (42779)	
55 Hospitalization/ (91123)	
56 Intensive Care Units/ (45436)	
57 exp. Life Support Care/ (8408)	
58 Operating Rooms/ and (acute* or critical* or emergenc* or intensiv* or trauma* or urgent*).mp. (1581)	
59 Respiratory Care Units/ (579)	
60 exp. Specialties, Surgical/ and (acute* or critical* or emergenc* or intensiv* or trauma* or urgent*).mp. (16202)	
61 Surgery Department, Hospital/ and (acute* or critical* or emergenc* or intensiv* or trauma* or urgent*).mp. (1066)	
62 ((acute* or critical* or emergenc* or intensiv* or trauma* or urgent*) adj2 (care or centr* or department* or hospital* or unit* or ward*)).tw,kf. (270869)	
63 ((acute* or critical* or emergenc* or intensiv* or trauma* or urgent*) and (intraoperative or operative or perioperative or postoperative)).tw,kf. (114703)	
64 ((burn* or cardi* or coronary* or heart* or respiratory*) adj2 (care or department* or room* or unit* or ward*)).tw,kf. (27819)	
65 ICU.tw,kf. (44322)	
66 life support.tw,kf. (10639)	
67 or/46–66 [Combined MeSH & text words for acute healthcare settings] (564082)	
68 and/28,45,67 [Combined concepts for opioids, chronic drug use, & acute healthcare settings] (2136)	
69 exp. animals/ not humans/ (4426250)	
70 68 not 69 [Exclude animal studies] (2116)	
71 (adolescent/ or exp. child/) not exp. adult/ (1302784)	
72 (adolescen* or child* or infan* or neonat* or p?ediatric* or youth).ti,jw. (1500398)	
73 70 not (71 or 72) [Exclude pediatric studies] (1845)	
74 (comment or editorial or news or newspaper article).pt. (1210379)	
75 73 not 74 [Exclude opinion pieces] (1810)	
76 (“1996 *” or “1997 *” or “1998 *” or “1999 *” or 200* or 201*).dt. (17023135)	
77 and/75–76 [date limit applied] (1410)	
78 limit 77 to (english or french) (1322)	
79 remove duplicates from 78 (1315)	

### Study assessment

Articles were identified through two phases. First, two authors (JD1 and JG) independently reviewed the titles and abstracts of all retrieved articles for study inclusion. Second, full texts of the selected articles were retrieved, reviewed and selected based on inclusion criteria. All steps were performed in duplicate, and in each phase, disagreements were resolved through discussion. In the case of unresolved matters, a third author (OGR) was involved. Reasons for exclusion of full text articles were recorded and displayed in a PRISMA diagram format.

### Quality assessment of studies

The methodological quality of each study was independently analyzed by two authors (JD1 and JG) using the Newcastle-Ottawa Quality Assessment Scale (NOS) for observational studies and the Cochrane Risk of Bias Tool for randomized controlled trials [24, 30]. Disagreements were resolved through discussion, and in the case of unresolved matters, a third author (OGR) was involved.

### Data analysis and synthesis

An inventory of supportive strategies was developed from the included studies. Descriptive analysis and data extraction of patient and study characteristics, and of supportive strategies and their outcomes were performed using standardized electronic data forms (Supplementary Appendix 1 for variables extracted). We analyzed all available data qualitatively and, when possible, aggregate analysis was performed [31]. For each study we extracted or computed the ratio of means between the intervention and usual care groups with 95% confidence interval [32]. These ratios were then pooled using the DerSimonian-Laird random effects method with an inverse variance weighting. To minimize bias due to confounding, results for RCTs were pooled separately from results for cohort studies and the two were not combined in one meta-analysis. Heterogeneity was assessed using the I-squared statistic with values greater than 50% considered to be “substantial” heterogeneity.

## Results

**Fig. 1:**
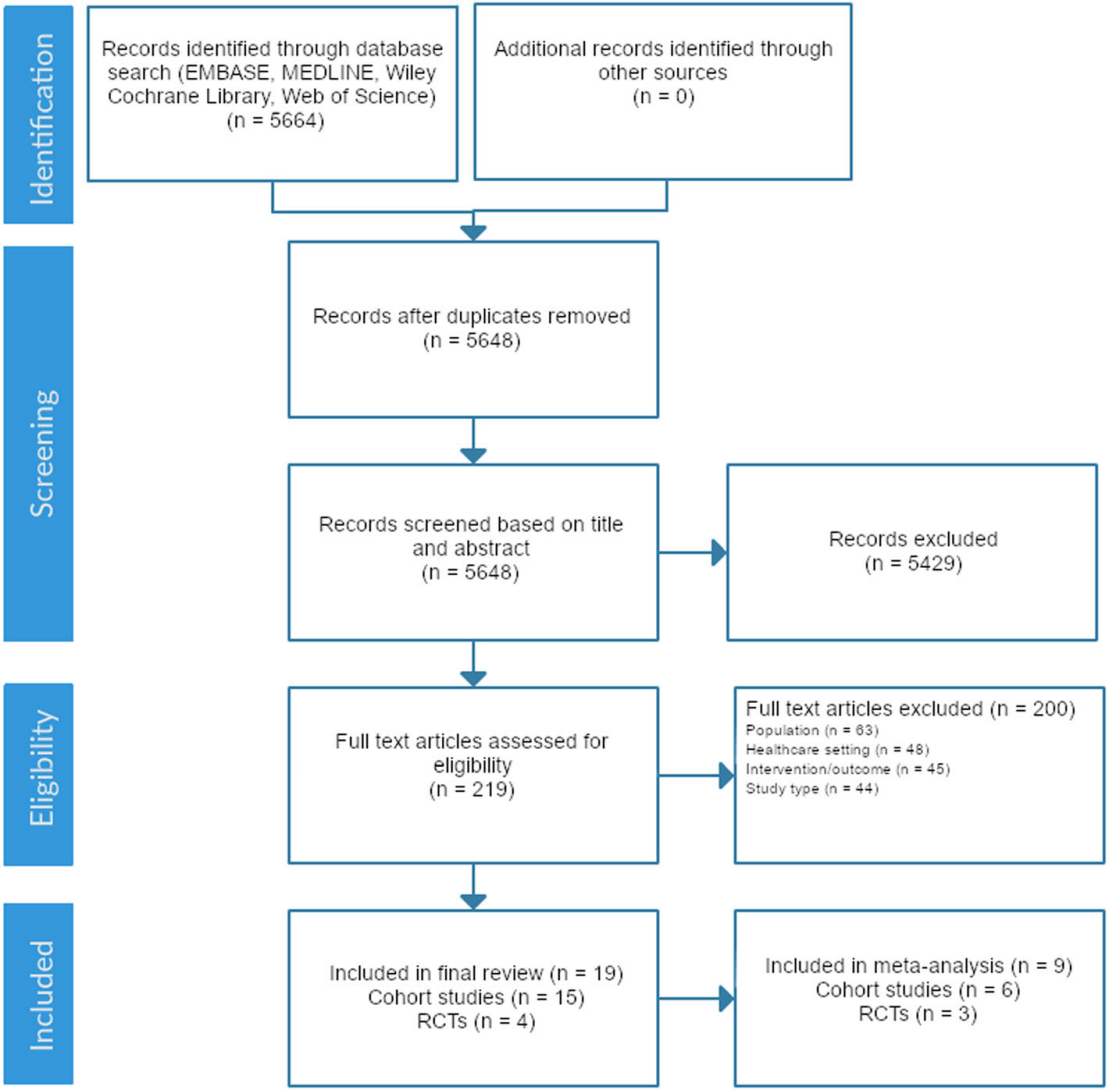
Flow diagram for study assessment

Our search yielded 5664 studies, of which 21 studies fulfilled our eligibility criteria (Fig. 1, Supplementary Appendix 2). These included 17 full-text articles and 2 abstracts, of which 15 were cohort studies and 4 were randomized controlled trials (Table 2). No additional articles were identified for inclusion from the reference list of included articles. Study quality is reported in Tables 3 and 4.

**Table 2:** Descriptive characteristics of included harm reduction intervention studies

Author and Year of Publication	Setting	# of Subjects	Study detail Inclusion criteria	Supportive Intervention Detail	Supportive Intervention Category	Outcomes	Follow-up Post Intervention
Cohort studies							
Alburaih (2018) [33]	ED	314	Retrospective multi-centre. Recurrent visits for pain.	ED-based pain contract (opioid treatment plan)	Support for pt. in pain.	# of ED visits	24-months
Alexandridis (2018) [34]	ED + Community	7200	Statewide database analysis Chronic pain pt. presenting to ED	Diversion control Naloxone policies Community education Provider education Support for pt. in pain Hospital ED policy Addiction treatment	Diversion control Naloxone policies Community education Provider education Support for pt. in pain Hospital ED policy Addiction treatment	Overdose mortality Overdose-related ED visits	22-months
Alexandridis (2019) [35]	ED + Community	7200	Retrospective Statewide database analysis Chronic pain pt. Subgroup of pt. on buprenorphine therapy	Diversion control Naloxone policies Community education Provider education Support for pt. in pain Hospital ED policy Addiction treatment	Diversion control Naloxone policies Community education Provider education Support for pt. with pain Hospital ED policy Addiction treatment	PDMP-derived counts of opioid prescriptions and buprenorphine	22-months
Allen (2016)^a^ [36]	ED + Community	13	Retrospective Chart review Pt with > 360 ER visits in 12 months Complex pain syndrome Problematic substance use	Comprehensive pain and addiction strategy referral from ED.	Support for pt. in pain	# of visits to ED Disposition of pt. after ED visit	52-months
Fulton-Kehoe (2015) [37]	Statewide	1809	Retrospective Statewide database analysis Pt with >= 1 paid claim for opioid Rx from ED.	Statewide Guideline for best practice implementation	Statewide Prescription Policies	Rates of non-methadone associated opioid poisonings	45-months
Ghobadi (2018) [38]	ED	19,751^c^	Retrospective Chart review Chronic pain (> 50 MEQ/d for >90d as outpatient)	Multi-ED opioid prescribing guidelines implementation	Statewide Prescription Policies	ED parenteral opioids ED oral opioids ED discharge prescription counts	12-months
Gugelmann (2013) [39]	ED	2462^b^	Prospective Pt receiving opioids in ED Subgroup analysis of pt. with chronic pain.	Multifacted educational program (round presentations, electronic notification, formal ED nursing staff education, journal clubs).	Provider Education	# of opioid discharge packs Change in opioid dispensing in pt. with RF for dependence	12-months
Hartung (2018) [40]	Statewide	N/A	Retrospective Medicaid administrative claims and enrollment data Pt with opioid Rx in ED. Stratification by MEQ dispensed.	Prior authorization for opioid Rx > 120 mg/d MEQ implementation.	Statewide Prescription Policies	Probability of opioid prescription above 120 mg MED	18-months
Jurecska (2012) [41]	ED	91	Retrospective Chart review Pt with > 3 ED visits in prior 3-Mos or 6 or > presentations in 6-Mo with chronic pain (defined as pain > 6 Mos).	Non-narcotic and adherence rates to narcotics policy implementation	Statewide Prescription Policies	Recurrent visits to ED	36-months
Kahler (2017) [42]	ED	243	Retrospective Chart review Pt with chronic pain Pt with >=6 ED visits per 12 Mo + at least 1 visit identified as primarily opioid-seeking behavior + case management for ED misuse.	Referral to free outpatient taper-to-abstinence pain management clinic.	Support for pt. in pain	# of ED visits # of PDMP opioid prescriptions # of individual opioid prescribers # of diagnostic tests	12-months
Maughan (2015) [43]	ED	N/A	Retrosepctive Database analysis through DAWN (Drug Abuse Monitoring Network) All ED visits involving opioid analgesic related harm (abuse or accidental)	Implementation of prescription drug monitoring program (PDMP)	Electronic Alert System	Rates of ED visits	84-months
Olsen (2016) [44]	ED	46	Retrospective + prospective Chart review Pt with > 3 ED visits in prior 6-Mo or > 6 ED visits in prior 12-Mo for a chronic painful condition. Inappropriate opioid prescription management	ED opioid prescription drug treatment plan in cooperation with primary care provider.	Support for pt. in pain	# fo ED visits # of opioid pills prescribed	6-months
Pace (2017) [45]	ED	529	Retrospective Chart review Acute pain Chronic pain (> 3 Mo)	Opioid prescribing pathway with framework for opioid prescription	Hospital ED policy	MEQ dose administered in ED # of IV/IM prescrpitions # of opioid prescriptions at discharge	6-months
Svenson (2007) [46]	ED	15	Prospective Chart review Pt with > 10 ED visits in prior 12-Mo for chronic non cancer pain.	ED organized care with non-opioid Rx and referral to primary care provider for opioid management.	Support for pt. in pain	# of ED visits # of outpatient clinic visits # of outpatient opioid prescriptions	12-months
Whiteside (2017) [47]	ED	29	Prospective open Feasibility study Subgroup analysis of ED pt. screened positive for risk of Rx opioid misuse in prior 6-Mo	ED-LINC: Emergency department longitudinal integrated care. Multidisciplinary case management: active care coordination and linkage, opioid guidelines, PDMP usage.	Support for pt. in pain Electronic Alert System Hospital ED policy	Feasibility of intervention Substance use and mental health scores # of ED visits	6-months
Randomized Controlled Trials							
Murphy (2017)^d^ [48]	ED	165	Multi-centric Non-blinded Pt with 5 or > ED visits in prior 12-Mo with > pain complaints or drug-seeking behavior. ED presentation > 50% related to pain. Economic evaluation (same cohort as Neven 2016)	Multidisciplinary case management with organized follow-up by case manager.	Support for pt. in pain	Total treatment cost differential	12-months
Neven (2016) [49]	ED	165	Multi-centric Non-blinded Pt with 5 or > ED visits in prior 12-Mo with > pain complaints or drug-seeking behavior. ED presentation > 50% related to pain.	Multidisciplinary case management with organized follow-up by case manager.	Support for pt. in pain	# of ED visits Odds of receiving an opioid prescription at ED discharge MEQ of opioid dispensed	12-months
Rathlev (2016) [50]	ED	40	Multi-centric Non-blinded Pt with 4 or > ED visits in prior 12-Mo with opioid use disorder (OUD) identified via SMS billing codes ED presentation related to acute pain.	Multidisciplinary case management development	Support for pt. in pain	MEQ prescribed at discharge MEQ administered in ED or inpatient Total medical charges # of ED visits # of ED visits with advanced imaging # of inpatient admission	12-months
Ringwalt (2015) [51]	ED	411	Pt with 11 or > ED visits in prior 12-Mo and chronic noncancer pain determined via chart & Rx review	Care linkage to primary care provider with plan for non-opioid based pain management.	Support for pt. in pain	# of prescriptions received from ED. # of ED visits	12-months

^a^Abstract only. ^b^ Subgroup analysis of pt. on opioids at ER presentation (pre=1512 and post=950). ^c^ Subgroup of chronic opioid use pt. pre + post. ^d^ Murphy (2017) [48] is an economic evaluation of the population and harm reduction strategy studied in Neven (2016) [49]. *MINI* Mini-International Neuropsychiatric Interview as per DSM-IV criteria, *Rx* prescriptions, *MME* Morphine milliequivalents (synonymous with mean morphine equivalent / MEQ), *ED* Emergency department, *PCP* Primary care provider, *RF* Risk factors, # Number, *Pt* pt., *Mo* Months, *d* Days. All studies listed were compared to usual care as defined as standard practice in the institution

See supplementary appendix 1 for full references

**Table 3:** Quality assessment of the studies included using the Newcastle-Ottawa Scale (NOS)

Author	Representativeness of exposed cohort	Selection of non-exposed cohort	Ascertainment of harm reduction	Outcome of interest absent at start of study	Comparability of cohorts	Assessment of outcome with independency	Adequacy of follow-up length
Alburaih (2018) [33]	*		*	*	**	*	*
Alexandridis (2017) [11]	*	*	*	*		*	*
Alexandridis (2018) [34]	*	*	*	*		*	*
Allen (2016) [36]	*	*	*	*		*	*
Fulton-Kehoe (2015) [37]	*	*	*	*	*	*	*
Ghobadi (2018) [38]	*	*	*	*		*	*
Gugelmann (2013) [39]	*	*	*			*	*
Hartung (2018) [40]	*	*	*	*	*	*	*
Jurecska (2012) [41]							
Kahler (2017) [42]	*	*	*	*	**	*	*
Maughan (2015) [43]	*	*	*	*		*	
Olsen (2016) [44]	*	*	*	*		*	*
Pace (2017) [45]	*	*	*	*		*	*
Svenson (2007) [46]	*	*	*	*		*	*
Whiteside (2017) [47]	*	*	*	*		*	

A maximum score of 9 is possible. A score of 0 to 3 represent low quality; 4 to 6 represent moderate quality; 7 to 9 represent high quality.

**Table 4:** Quality assessment of randomized controlled trials using the Cochrane Risk of Bias Tool

Author	Random sequence generation	Allocation concealement	Selective reporting	Other bias	Blinding of participants	Blinding of outcome	Incomplete outcome data	Final risk of bias
Rathlev (2016) [50]	Low	Low	Low	Low	High	Unclear	Low	High
Neven (2016) [49]	Low	Low	Low	Low	High	Low	Low	Mod
Murphy (2017) [48]	Low	Low	Low	Low	High	Low	Low	Mod
Ringwalt (2015) [51]	Low	High	Low	Low	High	Low	Low	Mod

A total of 9 categories of supportive strategies were identified (Table 5). Most studies addressed multiple supportive strategies simultaneously making separation difficult. This is reflected and detailed in both Tables 2 and 5. The 15 cohort studies included all assessed supportive strategies in a pre/post intervention model (Table 2). They either compared matched cohorts of different patients (*n* = 8) or cohorts of same patients before and after intervention (*n* = 7) in which each patient was his own control. Nearly all studies were performed in ED settings (*n* = 13). A single cohort study assessed mortality [11]. The four RCTs (Table 2) were all performed in the ED, with the comparator being usual care. Outcomes assessed included ED opioid prescriptions, ED discharge opioid prescriptions, hospital length of stay (LOS), and overdose and opioid related ED visits. All cohort and RCT studies were conducted in the US other than the cohort study by Allen et al. in Canada [36].

**Table 5:** Supportive interventions identified

Harm Reduction strategy	General Definition	Cohort	RCTs
Support for patients in pain	Support groups, case management and pain clinic vetting and referrals.	8	4
Hospital ED policy	Local practices to limit ED or inpatient OA prescribing and checking prescription drugs monitoring programs prior to prescribing	4	
Electronic alert system	Systems that alert providers to possible opioid abuse situations without mandating their use	4	
Provider education	Education of medical professionals in chronic pain treatment	3	
Statewide prescription policies	Practices or wide-ranging regulations to limit OA prescription within a legislative territory	3	
Addiction treatment	Opioid agonist therapies and policies supporting their use	2	
Community education	Promotion of public awareness of prescription opioid overdose	2	
Diversion control	Removal of unused medications and training of local law enforcement with OA diversion	2	
Naloxone policies	Promotion of the adoption of policies to disseminate the opioid antagonist naloxone to opioid users	2	

**Fig. 2:**
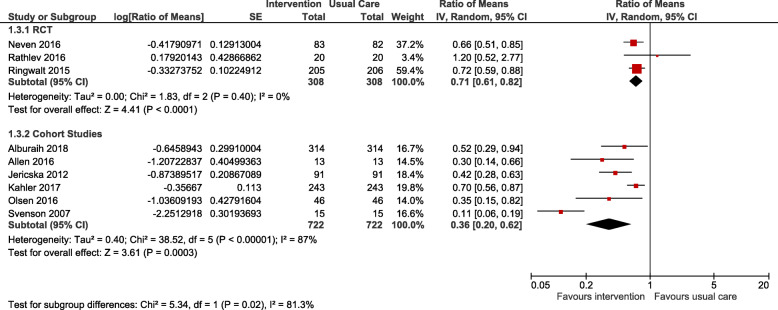
Meta-analysis of support for patients in pain supportive interventions for number of ED visits outcome

**Fig. 3:**
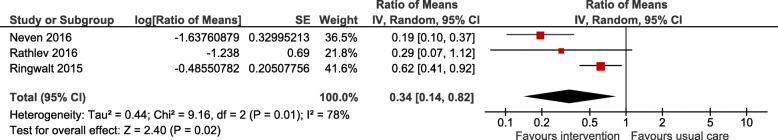
Meta-analysis of supports for patients in pain supportive interventions for ED discharge opioid prescriptions outcome

Six cohort studies and three RCTs were suitable for meta-analysis (Figs. 2 and 3). All studies included in the meta-analysis assessed outcomes for the “supports for patients in pain” supportive intervention. The specific interventions included: care linkage to primary care provider with non-opioid ED intervention plan (*n* = 2) and ED opioid intervention plan (*n* = 1), isolated opioid ED intervention plan (*n* = 2), comprehensive pain and addiction strategy referral (*n* = 2) and multidisciplinary case management (*n* = 3). Two outcomes (number of ED visits and number of ED discharge opioid prescriptions) were meta-analyzed, representing 1030 patients. The ED visits outcome refers to total number of visits to the ED after implementation of the strategy and was assessed in 9 studies (6 cohort, 3 RCTs) representing 1030 patients. The ED discharge opioid prescriptions outcome refers to the number of prescriptions dispensed at discharge of the patients from the ED and was assessed in 3 studies (3 RTCs) representing 308 patients. A significant reduction in number of ED visits for cohort studies (ratio of means 0.36, 95% with CI [0.20–0.62], I^2^ = 87%) and for RCTs (ratio of means 0.71, 95% with CI [0.61–0.82], I^2^ = 0%) was apparent. A significant reduction in number of ED discharge opioid prescriptions was noted for RCTs (ratio of means 0.34, 95% with CI [0.14–0.82], I^2^ = 78%). No patient-centered outcomes could be meta-analyzed. The studies duration ranged from 6 to 52 months.

## Discussion

This systematic review identified 11 supportive strategies for patients on long-term prescription opioids presenting to an ED with complications related to their opioid therapy. A pooled analysis of outcomes for “support for patients in pain” showed a clinically important decrease in the number of ED visits and ED discharge opioid prescriptions. Other supportive strategies could not be analyzed in a rigorous fashion and may be considered by healthcare providers until additional evidence becomes available.

Opioid use is an important and increasing problem in the US and Canada. Multiple supportive strategies for acute healthcare settings have been developed and studied, but the evidence had not been collated for an assessment of their impact. Most of the supportive strategies identified were from small, single center studies, and were too heterogeneous to be meta-analyzed or were infrequently studied (Table 5). While most reported positive results, a number of these are single-center studies with a small number of patients. These small studies often lack the scientific rigor or external validity to allow meaningful interpretation in a larger context, and to support widespread changes in practice [52].

We identified multiple studies with enough data to perform a meta-analysis for outcomes of the “support for patient in pain” strategy (Table 5). These studies were chosen due to their similarity in the coordinated care models used and the target populations. There was a clinically important decrease in system-related outcomes of ED visits and ED discharge opioid prescriptions for this strategy. For both outcomes, 3 RCTs were included with the most compelling data for ED visits due statistical significance and uniform data (I^2^ 0%), while ED discharge opioid prescriptions were significant but showed substantial heterogeneity (I^2^ 87%). The ED visits outcome was also supported by the meta-analysis of cohort studies that all trended in the same direction despite substantial heterogeneity (I^2^ 87%). As discussed above, “support for patients in pain” represent an aggregation of strategies individualized to a specific patient’s needs, which is widely different from other supportive interventions. These types of interventions are of course more resource intensive. Overall, the evidence demonstrates that the costs of treatment for opioid misuse and abuse are offset by the reduced health care costs [53, 54]. Murphy et al. provides the only economic analysis that indicates similar findings [48] and may support an economic incentive to their use in long-term opioid users without opioid use disorder. Furthermore, these interventions have clear evidence and support for patients with chronic non-cancer pain and opioid use disorder [55–57]. They are accordingly recommended by different international guidelines with recommendations [58]. Unfortunately, our findings are more complex in terms of interpretation by the nature of the outcomes used. Indeed, across all studies, there were only four instances of patient-related outcomes being evaluated. In these cases, the decrease in system-related outcomes were associated with unfavorable patient-related outcomes. Fulton-Kehoe indeed showed an increase in methadone poisonings as the number of opioid prescriptions and poisonings decreased [37] Alexandridis et al. was the only study with a favorable patient-related outcome, demonstrating lower overdose mortality related to healthcare professional education, but as a whole did not change the rate of ED visits [11]. This highlights concerns by experts that harm reduction strategies that focus on decreasing opioid prescriptions might actually contributed to unanticipated increases in avoidable deaths and overdoses [59] as patients seek out non-prescribed opioids to replace the previously prescribed opioids. The outcomes meta-analyzed may thus represent a poor proxy for appropriately impactful supportive strategies.

The other supportive strategies listed in Table 5 represent a combination of frequently recurring well-defined supportive strategies as well as composite terms representing supportive strategies referred to with different names across studies. This was determined through careful review of the detailed intervention performed in each study in order to reclassify them under umbrella headings. Unfortunately, precise definitions for each harm reduction strategy identified were not present in most studies. This limits our ability to both have homogeneous interventions under each harm reduction strategy. As such, based on the analysis of the interventions performed, most studies have multiple simultaneous harm reductions strategies employed. Accordingly, this limits the rigorous analysis of each harm reduction strategy independently.

In a similar fashion, there are no comparative studies of supportive strategies to inform which strategies may be superior, in which specific context, and where to direct organization and resources. Alexandridis et al. was the only study to include multiple well-differentiated strategies but analyzed them as independent variables despite a simultaneous implementation [11]. However, identifying a superior strategy may be of limited importance, as statistical superiority does not necessarily reflect the clinical reality in these complex patients. Indeed, the most appropriate strategy depends on multiple local factors such as individual patient’s specific needs and availability as well as access to resources. This highlights the complexity of assessing these process of care interventions for successful implementation and effectiveness of intervention. Such interventions may not lead to statistical or clinical significance in traditional outcomes (i.e., mortality) but have wider ranging benefits in care processes, workflow and resource optimization, as in the case and wide adoption of medical emergency teams (MET) [60].

### Strengths and limitations

While this study had several important strengths (i.e., breadth of scope, rigorously pre-defined methodology stretching across several medical domains, presence of patient advisors), several important limitations warrant discussion. First, important terms (i.e., long-term medical opioid therapy, opioid ‘abuse’ and misuse, harm reduction strategies) were heterogeneously defined across studies and may have been a barrier to study identification. Most importantly, the supportive strategies were overall poorly defined across studies. Despite a careful analysis of the interventions to regroup or reclassify them under umbrella terms, it was difficult to clearly identify separate supportive strategies in some studies. Accordingly, these studies then often used multiple supportive strategies simultaneously, which significantly limited our ability to have a rigorous analysis. This is reflected in the meta-analysis where the most important harm reduction strategy was analyzed, acknowledging that it may not be fully separated from other minor elements of the intervention that may be classified under another umbrella term. We attempted to mitigate these factors by independent screening by two authors to ensure the inclusion of all relevant studies and appropriately classify the supportive strategies. Second, the rate of study inclusion was only 0.4%. This was secondary to most identified studies either studied illicit drug use or poorly differentiated long-term opioid use without opioid disorder from opioid use disorder. We aimed to exclude opioid use disorder or abuse but were faced with a high degree of uncertainty in some cases. We thus decided to include studies only if they specifically referred to acute pain presentation in long-term opioid users even if there was mention of prescription opioid misuse, as long as there was some clear distinction between the groups. Given that there is a degree of conversion from appropriate use to abuse, we believe that this captures well this evolution in patients. Similarly, identifying what constitutes harm or complications from long-term opioid use proved challenging. Presentations other than overdose or without the attached opioid misuse label are often unrecognized as related to opioids. We had to assume that in the selected papers, the focus on patients being on opioid therapy means that there is a reasonable expectation that their presentation is related to opioid in some way. In our opinion, acute on chronic pain qualified as such, as it either represents hyperalgesia or under-treatment, both of which require a measured treatment approach. Third, the wide scope of some supportive strategies lead to difficult decisions for study inclusion. Indeed, a number of harm reductions were part of a package organized at a state level. It was difficult to separate the specific impact of each strategy, the impact on acute versus non-acute healthcare settings and to discern which studies dealt with patients on appropriate long-term opioid therapy. In these situations, we opted to include these state level studies if there was a well-described significant proportion of long-term opioid users, and if number of acute healthcare presentations was an outcome of interest. We do acknowledge that these studies reflect a very heterogeneous group in a lot of instances and limit the validity of the findings. This is not reflected well in the quality assessment of the cohort study who are technically for the most part of moderate to high methodological quality. The RCTs are for their part paradoxically at moderate to high risk of bias due to their design but represent a more homogeneous population. Fourth, most identified studies were from the US, limiting the generalizability of our findings to other jurisdictions that may have different policies and context that affect the outcomes of the identified supportive strategies. This is not surprising as the opioid epidemic was first recognized in the US, and many findings in the US are applicable across Canada and other high-income countries [1]. Finally, while we decided to include studies from 1996, all of the studies included are from the last 15 years. This is likely explained by the delayed recognition of the public health crisis from the opioid epidemic.

### Future directions

Our systematic review revealed that most of the studies have targeted patients presenting to the ED, with very little data on inpatient supportive strategies. This knowledge gap is reflected in the most recent Canadian guidelines for opioid use for chronic non-cancer pain, which do not address acute admissions in this population [58]. These guidelines do reflect the importance of a multidisciplinary approach in the chronic non-cancer pain population, which would be similar to the “supports for patient in pain” harm reductions strategy. Studying this harm reduction strategy for non-ED acute healthcare settings would strengthen the current body of evidence. Importantly, studying these strategies using patient-related outcomes such as mortality, quality of life and pain is of paramount importance, as opioid prescriptions and ED visits appear to be poor or misleading surrogate endpoints. Future policy work informed by these results would lead to better resource utilization through a shift from reactionary processes (i.e., ED visits) to preventative strategies that prevent acute healthcare presentations.

## Conclusion

We identified 9 supportive strategies for patients chronically prescribed opioids presenting to acute healthcare. The only harm reduction strategy that showed evidence of efficacy what “support for patients in pain” with clinically important decrease in the number of ED visits and ED discharge opioid prescriptions. Unfortunately, other supportive strategies were not evaluated in a rigorous fashion and may be considered by healthcare providers until additional evidence becomes available. These strategies have been studied almost exclusively in ED patients, and data on inpatient harm reduction is lacking and requires further study.

## Supplementary Information

**Additional file 1.** Variables extracted from included articles.

**Additional file 2.** References of included studies.

## Data Availability

The data generated or analysed during this study is available from the corresponding author upon reasonable request.

## Abbreviations

ED: Emergency Department
ICU: Intensive Care Unit
RCT: Randomized Control Trial
MET: Medical Emergency Team
