# Supplementary material for: Association between supportive interventions and healthcare utilization and outcomes in patients on long-term prescribed opioid therapy presenting to acute healthcare settings: a systematic review and meta-analysis
Source: BMC Emerg Med. 2021 Jan 29;21:17. doi: 10.1186/s12873-020-00398-9 (PMC7845034; doi:10.1186/s12873-020-00398-9)
Supplement: Supplementary file 1 — Additional file 1. Variables extracted from included articles. [file 12873_2020_398_MOESM1_ESM.docx]

Additional File 1. Variables extracted from included articles

Author

Year of publication

Harm reduction strategy

Comparator group

Setting

Single vs multiple centers

Study type

Number of patients recruited

Number of patients analyzed

Loss to follow-up

Age

Sex

Chronic pain

Presenting complication

Duration of study assessment

Opioid prescription outcome statistics

Overdose related ED visits outcome statistics

Opioid related ED visits outcome statistics

Hospital length of stay outcome statistics

Mortality (overall) outcome statistics

Linkage to resources outcome statistics

Quality of life outcome statistics
