# Supplementary material for: Association between supportive interventions and healthcare utilization and outcomes in patients on long-term prescribed opioid therapy presenting to acute healthcare settings: a systematic review and meta-analysis
Source: BMC Emerg Med. 2021 Jan 29;21:17. doi: 10.1186/s12873-020-00398-9 (PMC7845034; doi:10.1186/s12873-020-00398-9)
Supplement: Supplementary file 2 — Additional file 2. References of included studies. [file 12873_2020_398_MOESM2_ESM.docx]

Additional File 2. References of included studies

1. Alburaih A, Witting MD. Effectiveness of a Rural Emergency Department (ED)-Based Pain Contract on ED Visits Among ED Frequent Users. J Emerg Med. 2018;55(3):327-332.e1.
2. Alexandridis AA, McCort A, Ringwalt CL, Sachdeva N, Sanford C, Marshall SW, et al. A statewide evaluation of seven strategies to reduce opioid overdose in North Carolina. Injury Prevention. 2018 Feb;24(1):48–54.
3. Alexandridis AA, Dasgupta N, McCort AD, Ringwalt CL, Rosamond WD, Chelminski PR, et al. Associations between implementation of Project Lazarus and opioid analgesic dispensing and buprenorphine utilization in North Carolina, 2009-2014. Inj Epidemiol. 2019 Jan 21;6(1):2.
4. Allen MA. P008: Addressing chronic pain and problematic substance use of opioids in the emergency department: can a comprehensive framework for care work? Canadian Journal of Emergency Medicine. 2016 May;18(S1):S81–S81.
5. Fulton-Kehoe D, Sullivan M, Turner J, Garg R, Bauer A, Wickizer T, et al. Opioid Poisonings in Washington State Medicaid: Trends, Dosing, and Guidelines. Medical Care. 2015 Aug;53(8):679–85.
6. Ghobadi A, Winkle PJV, Menchine M, Chen Q, Huang BZ, Sharp AL. Reduction of Parenteral Opioid Use in Community Emergency Departments Following Implementation of Treatment Guidelines. Academic Emergency Medicine. 2018;25(8):901–10.
7. Gugelmann H, Shofer FS, Meisel ZF, Perrone J. Multidisciplinary intervention decreases the use of opioid medication discharge packs from 2 urban EDs. Am J Emerg Med. 2013 Sep;31(9):1343–8.
8. Hartung DM, Kim H, Ahmed SM, Middleton L, Keast S, Deyo RA, et al. Effect of a high dosage opioid prior authorization policy on prescription opioid use, misuse, and overdose outcomes. Subst Abus. 2018;39(2):239–46.
9. Jurecska DE, Peterson MA, Turgensen JN, Florea J. Pain: The continuing epidemic. J Pain Manage. 2012;5(3)(273–278):1.
10. Kahler Z, Musey P, Schaffer J, Johnson A, Strachan C, Shufflebarger C. Effect Of A “No Superuser Opioid Prescription” Policy On ED Visits And Statewide Opioid Prescription. Western Journal of Emergency Medicine. 2017 Aug 7;18(5):894–902.
11. Maughan BC, Bachhuber MA, Mitra N, Starrels JL. Prescription monitoring programs and emergency department visits involving opioids, 2004–2011. Drug and Alcohol Dependence. 2015 Nov;156:282–8.
12. Murphy SM, Howell D, McPherson S, Grohs R, Roll J, Neven D. A Randomized Controlled Trial of a Citywide Emergency Department Care-Coordination Program to Reduce Prescription Opioid-Related Visits: An Economic Evaluation. The Journal of Emergency Medicine. 2017 Aug;53(2):186–94.
13. Neven D, Paulozzi L, Howell D, McPherson S, Murphy SM, Grohs B, et al. A Randomized Controlled Trial of a Citywide Emergency Department Care Coordination Program to Reduce Prescription Opioid Related Emergency Department Visits. The Journal of Emergency Medicine. 2016 Nov;51(5):498–507.
14. Olsen JC, Ogarek JL, Goldenberg EJ, Sulo S. Impact of a Chronic Pain Protocol on Emergency Department Utilization. Bird SB, editor. Academic Emergency Medicine. 2016 Apr;23(4):424–32.
15. Pace C, Shah S, Zhang AX, Zosel AE. Impact of a chronic pain management pathway on opioid administration and prescribing in an Emergency Department. Clinical Toxicology. 2018 Aug 3;56(8):744–50.
16. Rathlev N, Almomen R, Deutsch A, Smithline H, Li H, Visintainer P. Randomized Controlled Trial of Electronic Care Plan Alerts and Resource Utilization by High Frequency Emergency Department Users with Opioid Use Disorder. Western Journal of Emergency Medicine. 2016 Jan 21;17(1):28–34.
17. Ringwalt C, Shanahan M, Wodarski S, Jones J, Schaffer D, Fusaro A, et al. A Randomized Controlled Trial of an Emergency Department Intervention for Patients with Chronic Noncancer Pain. The Journal of Emergency Medicine. 2015 Dec;49(6):974–83.
18. Svenson JE, Meyer TD. Effectiveness of nonnarcotic protocol for the treatment of acute exacerbations of chronic nonmalignant pain. The American Journal of Emergency Medicine. 2007 May;25(4):445–9.
19. Whiteside LK, Darnell D, Jackson K, Wang J, Russo J, Donovan DM, et al. Collaborative care from the emergency department for injured patients with prescription drug misuse: An open feasibility study. Journal of Substance Abuse Treatment. 2017 Nov;82:12–21.
